# Recent Advances in the Application of Bacteriophages against Common Foodborne Pathogens

**DOI:** 10.3390/antibiotics11111536

**Published:** 2022-11-02

**Authors:** Kinga Hyla, Izabela Dusza, Aneta Skaradzińska

**Affiliations:** Department of Biotechnology and Food Microbiology, Faculty of Biotechnology and Food Science, Wrocław University of Environmental and Life Sciences, Chełmońskiego 37, 51-630 Wrocław, Poland

**Keywords:** bacteriophages, food safety, phage biocontrol, foodborne pathogens, bacteriophage application, phage commercial products, foodborne illness

## Abstract

Bacteriophage potential in combating bacterial pathogens has been recognized nearly since the moment of discovery of these viruses at the beginning of the 20th century. Interest in phage application, which initially focused on medical treatments, rapidly spread throughout different biotechnological and industrial fields. This includes the food safety sector in which the presence of pathogens poses an explicit threat to consumers. This is also the field in which commercialization of phage-based products shows the greatest progress. Application of bacteriophages has gained special attention particularly in recent years, presumably due to the potential of conventional antibacterial strategies being exhausted. In this review, we present recent findings regarding phage application in fighting major foodborne pathogens, including *Salmonella* spp., *Escherichia coli*, *Yersinia* spp., *Campylobacter jejuni* and *Listeria monocytogenes*. We also discuss advantages of bacteriophage use and challenges facing phage-based antibacterial strategies, particularly in the context of their widespread application in food safety.

## 1. Introduction

Foodborne illness has been affecting people’s lives unceasingly, impacting human welfare and contributing to significant economic losses in many countries and populations.

Food products may become contaminated at different stages along the food chain including slaughtering, milking, fermentation, processing, storage, packaging or finally consumption of the product [1]. The most popular strategies for elimination of the pathogens are implementation of high standard hygiene procedures, rational running of the process line, and use of biocides and disinfectants [2]. However, currently applied methods for elimination of foodborne pathogens are unreliable. For instance, use of steam, dry heat or UV light leads to changes in organoleptic properties of the product. Moreover, there are some limitations in the use of certain antimicrobial approaches, in particular products such as fresh fruits, vegetables and ready-to-eat (RTE) products. A major problem is that extensive use of sanitizers leads to the development of microbial resistance [1].

Bacteriophages, or phages for short, are viruses that selectively infect and replicate in bacterial cells. Due to their unique properties, phages have been perceived as promising tools in combating bacterial pathogens not only in human treatments (phage therapy) but also in various industrial fields. This includes the food production sector in which, for the sake of the consumer’s safety, all implemented antimicrobial procedures must be selected with special care. In contrast to routinely used antibacterial approaches, phage application does not change the properties of the product, viruses may be applied on a variety of matrices and the problem of resistance may be overcome much more easily compared to chemical antibacterials [3,4,5]. Phages are isolated from a variety of foods, which indicates their natural contact with humans with this route [6]. Furthermore, phage-based strategies are cost-effective and consumer-friendly, making them an important alternative to standard antibacterial procedures.

The ability of bacteriophages to effectively eliminate foodborne pathogens has been reported in numerous scientific articles [7,8,9,10]. Since the latest years have brought a significant increase in bacteriophage studies, in this review, we summarize the recent findings regarding efficiency of phages against the main food-related pathogens and potential application of these viruses in approaches of microbiological control in food production. We believe that ensuring safety of food products is a key global concern, and searching for novel solutions that will allow high standards of food production to be maintained is urgently needed. In this aspect, the use of phages as tools providing safety for consumers should be seen as an important alternative to currently applied methods.

### 1.1. Bacteriophages

Bacteriophages are the most ubiquitous biological entities on Earth, with an estimated total global population of 10^31^ particles [11]. They are also known as phages, from the Greek *phagein* meaning “to eat” [12]. They can be found in a variety of ecosystems, including extreme environments, such as the Sahara desert, hot springs and cold polar waters [13]. They are characterized by a great diversity in structure, size and organization of the genome [14].

Bacteriophages were first mentioned in 1896, when the British bacteriologist Ernest Hankin reported unusual antibacterial activity in the waters of the Yamuna and Ganga rivers. Furthermore, he suggested that this unknown factor inhibited the development of the epidemic of cholera caused by *Vibrio cholerae*. Notably, his hypothesis has never been confirmed by the scientific community [15]. The researcher who first observed translucencies on a bacterial lawn caused by bacteriophages was Frederick Twort. However, at that time he considered it to be the action of a “transmissible vitreous transformation”, the amount of which increased after the death of the cell. In 1917, the French-Canadian microbiologist Félix d’Herelle published results of his research, describing the phenomenon of bright “zonas”, which he eventually called plaques. He was also the first to propose the hypothesis that the translucencies could be caused by a virus that parasitizes bacteria. He called it a “bacteriophage” [13].

One of the most characteristic features of bacteriophages is their high specificity, regarding one species, or a specific strain of bacteria they infect [14]. This high specificity is based on selective binding of the virus receptor with the ligand at the bacterial surface. Proteins, polysaccharides, lipopolysaccharides (LPS) and carbohydrate moieties as well as outer membrane proteins, pili and flagella may be used by phages as keys to the entrance to bacterial cells [16]. Bacteriophages multiply with two basic replication cycles. In the lytic cycle, once the genetic material of the phage is inside the relevant host, it is replicated with the molecular apparatus of the bacterium. In a relatively short time after phage penetration, new virions are assembled and released to the environment. This sequence of molecular events naturally leads to lysis of the bacterial cell [17]. Bacteriophages amplifying with the lytic cycle are called lytic or virulent phages [18]. In the lysogenic cycle, performed by temperate phages, nucleic acid of the virus integrates with the bacterial genome and multiplies with the host as a prophage. However, as a result of unfavorable changes in environmental conditions, the phage can revert to the lytic cycle [13]. Clearly, due to the lack of lytic activity, temperate phages are not relevant candidates for practical use.

Bacteriophage efficacy in disrupting bacterial cells is an excellent feature for their use as microbiological tools in antibacterial strategies. However, within years of phage application, other characteristics of bacterial viruses have been perceived as beneficial, particularly compared to standard chemical antibacterial agents. Bacteriophages recognize and infect only a particular bacterial host and therefore they do not affect the natural microflora of the organism. As a result, they are better tolerated by the human body than antibiotics and thus are considered a safer treatment option [19,20]. Moreover, frequently, a single dose of bacteriophage preparation is sufficient to achieve the therapeutic effect [21,22]. This results from the unique phage ability of “auto-dosing”, which means that phages are capable of increasing in number specifically where their hosts are located [23]. Bacteriophages are inhabitants of humans as they are found in the respiratory or digestive tract. It is thought that the first viruses enter the intestines within 4 days of birth [20]. This natural contact with phages also indirectly supports the safety of the intended phage application. It is not without significance that, compared to antibiotics, phage acquisition is easier, faster and cheaper [24]. For applications in food safety of particular importance is that phages do not impact organoleptic, rheological and nutritional properties of the product [25].

There are surely also limitations of phage use, such as the narrow spectrum of activity, which may be a serious obstacle in production of universal preparations intended for use on a large scale, or development of phage-resistant strains. However, both limitations may be overcome by the use of phage cocktails, which may be composed of phages with different specificity, broadening the lytic spectrum of the preparation, or with phages with similar activity so that when bacteria acquire resistance to one phage from the preparation they likely remain susceptible to another [24,26]. It is noteworthy that the effectivity of phage cocktails is multifaceted and other factors, including the mobilization of virulence or antibiotic resistance genes or phage coinfections have to be considered [27]. Limitations of phage application are discussed more specifically later in this review.

### 1.2. Foodborne Pathogens

The first food infections were reported in the 5th century BC, when it was observed that illnesses occurring at that time may be related to the consumed food [28]. Since then, scientists have shown that pathogens that contribute to food contamination include viruses, parasites and bacteria of which the latter are considered the most common cause of foodborne infections [29]. Among the bacterial species of utmost importance are frequently listed *Salmonella* spp., *Listeria monocytogenes*, *Escherichia coli* and *Campylobacter jejuni* [30,31].

Bacterial food-related pathogens are mainly mesophilic micro-organisms tolerating temperatures in the range of 20–45 °C, so they can easily survive in the human body. Of note, some pathogens, e.g., *Y*. *enterocolitica*, persist at temperatures lower than 10 °C, which entails the need to adjust preventive methods to remove potentially occurring bacterial cells. Production of spores by many bacterial species also hinders decontamination.

However, one of the most significant challenges is the ability of pathogens to form a biofilm structure [28]. In a biofilm, cells are embedded in the extracellular polymeric substance (EPS), also known as the extracellular matrix, providing micro-organisms resistance to environmental factors [32]. Food is a particularly favorable matrix for biofilm development. In the food production sector, biofilm structures may form directly on food products as well as on equipment that may come into contact with food [33]. Foodborne pathogens able to form biofilms are of particular concern for food producers as the use of disinfectants and other antimicrobial agents is inefficient due to the limited penetration of the product within the structure [34]. Even if the biofilm is cleared once, the contamination likely returns [33]. Many foodborne pathogens, e.g., *L. monocytogenes*, *S. enterica*, *C. jejuni* and *E. coli*, are able to form biofilms, so implementing antimicrobial strategies developed to efficiently remove these highly resistant structures is urgently needed.

There are two routes for the development of a foodborne infection. The first route, which is referred to as intoxication, is that a pathogen on the food surface or inside the food product produces a toxin, which then enters the organism with the meal and affects its metabolism. In the second case, a pathogen that has directly entered the digestive system with food is able to adapt and multiply within cells [28].

The most comprehensive data related to food infections date back to 2016 when the EFSA (European Food Safety Authority) published a summary report on trends and sources of zoonoses, zoonotic agents and food-borne outbreaks in the preceding year [35]. According to presented data, in 2015, there were 45,874 cases of food-related illness within the countries of the European Union. The number of outbreaks in which two or more people were infected after consuming the same food was 4362 of which 33.7% were caused by bacteria, mainly *Campylobacter* spp. and *Salmonella* spp. Reported foodborne infections were mainly related to contamination of products of animal origin, such as pork and eggs, but also other products as shellfish, milk, and fish [28]. In the same year, the World Health Organization (WHO) estimated the number of foodborne outbreaks across the globe. A total of 31 infectious agents were identified to contribute to the illnesses of 600 million people and the deaths of 420,000 people worldwide. Both reports conclude that special attention should be paid to food safety at every stage of production in the so-called “farm to fork” approach [36].

**Figure 1:**
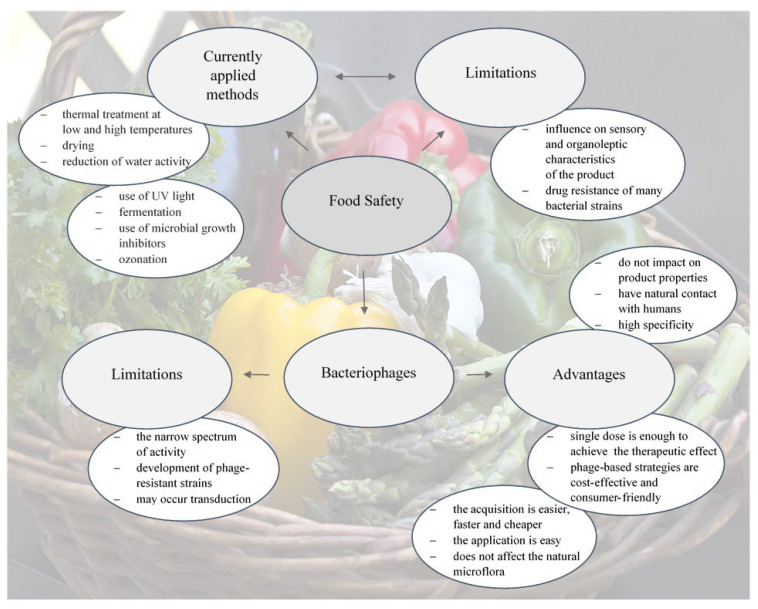
Currently used antimicrobial methods in food safety compared to bacteriophage application.

The possibility of food contamination depends on numerous factors, such as the type of the product and the method of its production, the water content in food, or the amount of accessible oxygen during the process which inhibits development of anaerobic bacteria but at the same time creates conditions for development of aerobic strains. There are various methods which are used as a standard to eliminate undesirable bacteria. These include thermal treatment at low (chilling, freezing) and high temperatures (pasteurization, sterilization), drying, reduction of water activity, fermentation, use of microbial growth inhibitors or ozonation. However, use of the available sanitization methods frequently influences sensory and organoleptic characteristics of the product (Figure 1) [37].

It is worth emphasizing that a number of system procedures have been developed and implemented to maintain standards of safety within food processing. These include good manufacturing practices (GMP), sanitation standard operating procedures (SSOP) and hazard analysis and critical control points (HACCP). The latter is the most widely used strategy in maintaining production safety [38]. Nevertheless, despite implementing different safety protocols, the final products must still be tested for microbial contamination to ensure consumer safety and reduce the risk of disease [39].

Especially noteworthy is the problem of the industrial breeding of livestock, particularly on large farms in which there is an increased risk of microbial contamination and eventually a disease outbreak due to hindered waste management control. It is commonly known that abuse of antibiotics in husbandry has become a relevant global problem. The amount of these drugs applied in farming exceeds 50 million kilograms each year worldwide [40]. Replacement of antibiotics is a critical challenge as abundant and virtually uncontrolled release of these medicaments into the environment contributes to the rapid development of resistant bacterial strains. Considering the demanding environment of livestock farms and the specificity of animal breeding, development of new preventive methods against microbial contaminations is critically needed.

### 1.3. Salmonella

*Salmonella* is a rod-shaped, Gram-negative bacterium belonging to the Enterobacteriaceae family. It is the cause of one of the most common foodborne diseases—salmonellosis. The illness usually manifests with abdominal pain, vomiting, diarrhea, fever and headache. Based on the EFSA report from 2020, the pathogen is transmitted through the soil, water, feed, and feces, among others. Bacteria are typically found in meat and animal products, which become the source of infection for humans. Chickens are the most frequently infected animals, followed by cattle, turkeys, pigs, ducks, and geese. The most common cause of infection are the two serotypes *Salmonella* Enteritidis and *Salmonella* Typhimurium [41].

The potential of bacteriophages in combating *Salmonella* infections has been confirmed in numerous studies [25,42,43,44]. A recent example is the research of Yan et al. (2020), who investigated the efficacy of the LYPSET phage in elimination of *S. enterica* in food products. The experiments were carried out using milk and lettuce stored at two temperatures, 4 °C and 25 °C. Samples contaminated with the pathogen were treated with phage lysate and the number of bacteria was determined. In the case of milk samples, application of the phage preparation allowed the number of bacteria to be reduced by 2.07 log CFU/mL at 4 °C and by 3.67 log CFU/mL at 25 °C with an MOI = 1000. When the MOI was 10,000, the activity of phages was slightly higher as the number of bacteria decreased by 2.19 log CFU/mL at 4 °C and 4.33 log CFU/mL at a temperature of 25 °C. For lettuce samples only MOI = 10,000 was tested. The number of *Salmonella* cells decreased by 2.2 log CFU/mL at 4 °C and by 2.34 log CFU/mL at 25 °C. The results confirmed that application of phages may be a promising approach in combating *Salmonella* contamination in food [45].

In similar studies of Islam et al. (2019) the effect of the phage cocktail composed of three phages, LPSTLL, LPST94 and LPST153, on *S.* Typhimurium and *S.* Enteritidis and their mixture in milk and in chicken meat was investigated. As in former studies, the experiments were performed at temperatures of 4 °C and 25 °C. The results showed that the number of *S.* Typhimurium cells in milk was reduced below the detectable limit (<1 CFU/100 μL) after 3 h and 6 h at 4 °C with MOI of 10,000 and 1000, respectively. For the mixture of *Salmonella* almost complete elimination of bacterial cells in milk was observed after 6 h and 12 h at 4 °C with MOI of 10,000 and 1000, respectively. Of note, for a single *Salmonella* strain or a mixture of *Salmonella* strains, the viable counts declined completely after 6 h (MOI = 10,000) and 24 h (MOI = 1000) at 25 °C. Similarly, for the chicken meat, there was complete elimination of bacteria after 3 h at 4 °C and 3 h and 6 h at 25 °C for MOI of 10,000 and 1000. Furthermore, the authors tested the effect of bacteriophage cocktail on biofilm structures formed by *S*. Typhimurium and the mixed culture of *S*. Typhimurium and *S*. Enteritidis. The experiments were performed using a 96-well plate and a stainless steel surface. Biofilm structures were formed and then treated with preparations with phage titer of 7 log PFU/mL and 8 log PFU/mL. After 24 h, biofilm reduction was determined for both matrices. In the case of the 96-well plate, reduction of *S*. Typhimurium biofilm structure was 48.3% and 63.25% for both tested phage titers. In analogical experiments with mixed biofilm the decrease was 44.28% and 63.25%. When using the steel surface and a single *S*. Typhimurium strain, the biofilm decreased by 5.5 log and 6.42 log for respective titers 7 log PFU/mL and 8 log PFU/mL. For a mixed biofilm, reduction of 44.28% and 51.17% compared to the control with no phage was achieved [46]. The results indicated that phage cocktails may be potentially used as biological control agents against *Salmonella*, including the removal of *Salmonella* biofilm structures from food-related equipment.

In another study, the potential of the SE07 phage specific to *S*. *enterica* isolated from chicken and beef meat intended for sale was tested. The effects of the phage in reducing *Salmonella* contamination was evaluated for a variety of foods, including fresh eggs, beef, poultry meat and fresh fruit juice. A significant reduction in the number of pathogens was noted after 12 h of the experiment; however, after the following hours the further decline of the bacterial count was negligible. In the example of beef, the number of bacteria decreased from the initial concentration of 4.23 log CFU/mL to 2.32 log CFU/mL after 12 h following phage application. Similarly, in the case of the chicken sample, the bacteria count dropped from 4.16 log CFU/mL to 2.34 log CFU/mL in the 12th hour of the experiment [47].

SalmoFresh™ (Intralytix, Columbia, SC, USA) is a bacteriophage preparation against *Salmonella* spp. which has been granted GRAS (Generally Recognize As Safe) status by the US Food and Drug Administration (FDA, 2013; Intralytix, 2015). According to information provided by the producer the product is specifically designed for treating foods that are at high risk for *Salmonella* contamination. Red meat and poultry in particular can be treated prior to grinding for significant reductions in pathogen count [48]. The preparation is composed of six phages targeting different serotypes of *Salmonella* spp. In the studies of Zhang et al. (2019), the surfaces of lettuce, mung bean sprouts and its seeds were covered with SalmoFresh, whereas the control group was washed with chlorinated water. In an additional experimental group, the food products were treated with a mixture of both chlorinated water and the phage preparation. The findings demonstrated the effectiveness of the cocktail in reducing *Salmonella* contamination on lettuce and sprouts as bacterial counts decreased during storage of the products by 0.76 log CFU/g and 0.83 log CFU/g, respectively. The results were inconclusive in the case of seeds as there was exponential growth of bacteria observed after their germination. Surprisingly, the most effective method turned out to be combined use of chlorinated water and a bacteriophage cocktail, which gave satisfactory results in all trials [49].

A relatively novel application of bacterial viruses is their use as components of detection systems for different bacteria. Minh et al. (2020) developed a method for the rapid detection of *Salmonella* using NanoLuc reporter phages. The gene of luciferase was introduced with homologous recombination downstream of the main capsid protein sequence. Bacteria were incubated with modified phages SEA1.NL and TSP1.NL for two hours and their presence was evaluated based on the luminescence production. A combination of the two phages provided the best results since the TSP1.NL phage gave high intensity of luminescence, while SEA1.NL showed high specificity. This method has been referred to as PhageDx for *Salmonella*. PhageDx has been proved to work flawlessly for pure cultures, and the question arose whether it could be effective for food matrices. Therefore, in subsequent studies, possible application of the method in detection of *Salmonella* contamination on ground turkey meat and a powder infant formula has been evaluated. PhageDx proved to be effective for both matrices since no false positive results were noted [50]. This suggests that application of bacteriophages in food safety may go beyond direct elimination of bacteria and phages can be potentially important tools in preventive strategies including microbiological detection systems.

### 1.4. Escherichia coli

Although *E. coli* is a natural inhabitant of the intestinal microflora of all mammals, at the same time it is the cause of many serious intestinal and extraintestinal diseases, including infant meningitis or sepsis [51]. There are two main groups of pathogenic strains of *E. coli*. The first is diarrheal *E. coli*, also known as enteric pathogenic *E. coli* (IPEC), which causes diarrhea or intestinal flu. This group includes well-known pathogens such as enterotoxigenic *E. coli* (ETEC), enteroinvasive *E. coli* (EIEC), enteropathogenic *E. coli* (EPEC) and shigatoxigenic or Shiga toxin-producing *E. coli* (STEC) with the subgroup of enterohemorrhagic *E. coli* (EHEC). The second group is extraintestinal pathogenic *E. coli* (ExPEC), which contributes to infections outside the intestines [52]. The bacterium is highly contagious as even a small number of bacterial cells may cause a disease. It is transmitted mainly by food and contaminated water [28].

In a recent study, Vengarai Jagannathan et al. (2021) investigated potential application of a bacteriophage cocktail in reducing the growth of pathogenic *E. coli* O157:H7 on food products. Fresh spinach leaves were rinsed for 10 min with sterile drinking water loaded with a mixture of *E. coli* and bacteriophages (MOI 2:3). Compared to the control group in which vegetables were dunk-washed with a water suspension of bacteria, a reduction in pathogen count of 99% was achieved [53].

Mangieri et al. (2020) used bacteriophages specific to STEC to reduce bacterial contamination of fresh cucumbers. A phage cocktail composed of three bacteriophages was tested on vegetables at two temperatures of 25 °C and 4 °C for 24 h. The number of pathogenic bacteria was reduced by 1.16 log CFU/g after 6 h and 2.01 log CFU/g after 24 h at a temperature of 4 °C and 1.97 CFU/g after 6 h and 2.01 log CFU/g after 24 h at 25 °C. The authors stated that phage cocktails may be potentially used in controlling bacterial contamination in fresh vegetables [54].

These observations were confirmed in another study by Dewanggana et al. (2022), who investigated the potential use of bacterial viruses to eliminate ETEC from different food products (chicken meat, fish meat, cucumber, tomato, lettuce). Food samples were rinsed with *E. coli* and then the phage lysate was used to remove the contamination. The samples were incubated at 4 °C and the number of bacteria was determined after 24 h and 6 days. For all food products a more significant effect was observed after 6 days of incubation. The highest efficiency in reducing *E. coli* was observed for the chicken meat as the number of bacteria decreased by 80.93% and 87.29% at days 1 and 6, respectively. The effect was found to be the weakest for lettuce as bacterial contamination was reduced by 46.88% and 43.38% for both tested temperatures [55].

Importantly, phages are also effective in removing the biofilm structure formed by *E. coli* on food. The influence of AZO145A bacteriophage on *E. coli* biofilms on beef was investigated by Wang et al. (2020). Meat treated with ε-polylysine was used as a control. The use of both phage and ε-polylysine showed beneficial effects in reducing biofilm transmission between pieces of meat. The elimination of biofilm structure was comparable for both disinfectants as phages reduced the bacterial count by 3.1 log CFU/coupon and ε-polylysine 2.9 log CFU/coupon. However, it was found that without increasing the doses of both preparations, biofilm reappeared in the trials. Such observations notwithstanding, AZO145A phage may be considered a promising agent in combating *E. coli* in meat products [56]. 

Choi et al. (2021) used a novel approach in reducing *E. coli* on raw beef meat. The authors immobilized bacteriophages on the surface of the polymer film used as a standard for food packaging. The objective of the study was to develop a material which would be safe for use and at the same time would provide antibacterial safety. The bacteriophage T4, which is probably the best known representative of phages specific to *E. coli*, was covalently attached to a polycaprolactone (PCL) film. Then, portions of raw beef contaminated with *E. coli* were packed in the packages with the phage. The bacterial growth was measured after 30 min and compared with the standard packages with no phage. The reduction of bacterial population was 2.44 log compared to the control group, demonstrating the possibility of using phages in systems of food packaging [57].

### 1.5. Listeria

*Listeria monocytogenes* is a Gram-positive, anaerobic, rod-shaped bacterium [58]. It is a foodborne zoonotic pathogen, which means that it is naturally transmissible from vertebrate animals to humans. In humans it causes listeriosis, the symptoms of which include encephalitis, meningitis, sepsis and gastrointestinal disorders. It is particularly dangerous for pregnant women since it may lead to miscarriages [59].

The recent findings confirm that bacteriophages may show high activity against *L. monocytogenes* strains found in meat and meat-derived products. Importantly, it has been observed that they do not have a detrimental effect on the natural microflora of the consumers. Moreover, phages have also been proven to be stable under refrigerated storage [60]. Undoubtedly, the aforementioned features are among those that have led to the extensive development of *Listeria* targeting commercial bacteriophage products.

Currently available phage preparations against *Listeria* include Phage LM-103a, Phage LMP-102a, Phage Ply511, ListShield (all of them Intralytix, USA), and Listex P-100 (Micreos Food Safety B.V.; The Netherlands) [61,62]. The effectiveness of these preparations in controlling *Listeria* contamination in different food products has been confirmed in several studies.

ListShield (formally known as LMP-102) was the first phage-based preparation to receive FDA approval, in 2006, for direct application on meat and poultry products that meet the ready-to-eat definition [63]. ListShield is a cocktail composed of six bacteriophages with high lytic activity against *Listeria* strains. It was shown that it significantly reduces the presence of pathogens in various types of RTE foods, with the efficiency of 82–99% [64]. Ishaq et al. (2022) reported that in the case of smoked salmon, the use of ListShield completely inhibited the growth of *L. monocytogenes* on naturally contaminated samples as well as those infected in an in vitro experimental model. Importantly, there were no organoleptic changes in the tasted samples, which is important from the perspective of the consumers. Moreover, ListShield has also been found to have bactericidal properties on surfaces, which may be of great importance in the context of protecting, for example, processing plants in the food industry against *Listeria* contamination [3].

Shortly after the ListShield approval, another *Listeria*-targeting preparation, Listex P100, was given GRAS status by the FDA and a positive opinion by the EFSA, which stated that the product is not only safe but also effective [65]. Listex P100, is composed of six *L. monocytogenes* specific phages and it is recommended for the reduction of bacterial contamination on RTE products of animal origin [66]. The product showed high specificity in trials performed at different temperatures. The largest decrease in bacterial count achieved was 4.44 log CFU/mL when the inoculum count was 3 log CFU/g [64]. Importantly, Listex P100 has also been proved to be effective in reducing the biofilm structure formed by *L. monocytogenes* on stainless steel with a reduction of 3.5–5.4 log CFU/cm^2^ compared to a control group in which no antibacterial agent was applied [67]. Compared to standard antibacterials, such as nisin and sodium lactate, Listex P100 has been proved to be more effective in eliminating *L. monocytogenes* on ready-to-eat, sliced ham [68]. Notably, former observations were confirmed that application of the preparation does not affect the organoleptic properties of the product [66].

Going beyond products of animal origin, potential application of phages in combating *Listeria* contamination on fruits has also been investigated. Phages were administered by injection into the pulp of contaminated melons and apples or evenly spread on the surface of the fruits. Bacterial contamination in melons decreased by 4 log units compared to the control group in which fruits were treated with nisin. Notably, in the case of apples, the amount of bacteria declined by only 0.4 log units compared to bacteriocin treated products. Furthermore, the effectiveness of nisin should be noted, as on fruits protected with this preparation the number of *Listeria* declined by 6 log units in the case of melon and by 2 log units in the samples with apples compared to the control group in which no disinfectant was applied [69].

### 1.6. Campylobacter

*Campylobacter jejuni*, one of the best known representatives of the Campylobacteraceae family, is one of the most important foodborne pathogens. It naturally inhabits the intestines of birds and mammals. However, in particular conditions, it may cause gastroenteric infections called campylobacteriosis, and hence *C. jejuni* is among the most common causes of zoonotic illnesses worldwide [28]. Infections are frequently caused by drinking contaminated water or raw milk and eating contaminated meat, especially chicken or beef, and other animal-derived products. The most common symptom of infection is diarrhea, but infected people may sporadically develop secondary diseases, such as Guillain–Barré or Miller–Fisher syndrome [70].

Thung et al. (2020) explored the potential use of bacteriophages as microbiological tools to control *C. jejuni* contamination in chicken meat and mutton. The meat was sliced and infected with bacteria, then incubated and sprayed with the phage preparation. The samples were stored at 4 °C for 2 days and the amount of *C. jejuni* was determined. The authors observed a decrease of bacterial number by 1.68 CFU/g and 1.70 CFU/g for chicken meat and mutton, respectively. These findings suggest the possibility for use of bacterial viruses in strategies against *C. jejuni* in meat products [71].

This hypothesis was supported by the results of another study in which two phages, Φ7-izsam and Φ16-izsam, were used to counteract the development of antimicrobial-resistant *C. jejuni* in poultry. First, animals were tested in terms of natural infections with the bacterium. Then, uninfected birds were orally given a *C. jejuni* suspension. Bacteriophages were provided to the animals prior to slaughter. Compared to the control group, bacterial counts in cecal content of animals treated with phage preparations were significantly reduced. The number of *C. jejuni* decreased by 1 log CFU/g and 2 log CFU/g for two groups of animals in which the phage preparation was given at different time points [72].

In a similar study of Richard et al. (2019), bacterial viruses were used to control *C. jejuni* in broiler chickens. Four days after the birds were infected, bacteriophages CP20 and CP30A were orally administered to the animals. The chickens were sacrificed every 24 h and *Campylobacter* counts in the intestinal lumen were determined. As a result, a remarkable reduction in a bacterial number in phage-treated groups was observed. The most significant effect was noted 2 days after the bacteriophage application, as the bacterial number decreased by 2.4 log CFU/g compared to the control group of infected, untreated animals. An important objective of the study was to investigate the influence of the phage treatment on the gut microflora of the animals. The results indicated that bacteriophages did not affect birds’ microbiota, which supports previous observations regarding the safety of phage treatment [73]. Of note, recent studies on bacteriophages of different specificity showed that phage application influences the microbiological balance in birds’ intestines [74,75,76,77]; however, reliably answering whether this influence is harmful or harmless for the animals requires further, comprehensive research.

The efficacy of bacteriophages in eliminating *C. jejuni* in chicken meat was also confirmed in the studies of Zampara et al. (2017). The authors isolated phages capable of reducing *C. jejuni* at a chilled temperature and then created a phage cocktail composed of two phages with the highest lytic spectrum. The cocktail reduced the number of bacteria by 0.73 log units compared to the control group. Remarkably, the results confirmed that phages may be used in protecting the chicken meat against *C. jejuni* also at lower temperatures [78].

It is noteworthy that campylophages, compared to other bacterial viruses, have some features which make their application difficult. Żbikowska et al. (2020), in a recent review on potential use of phages in the poultry industry, listed in this regard: (i) the problems associated with the optimization methods for phage isolation, propagation and purification; (ii) difficulties with appropriate selection of phage candidates for application due to the significant differences between *Campylobacter* phages within groups, even they are genetically very similar; (iii) evidenced phage resistance of *Campylobacter* after phage treatment; and (iv) the cost of production [79]. Notwithstanding these limitations, phage treatment of *Campylobacter* infections remains an appealing option.

### 1.7. Yersinia

*Yersinia enterocolitica* is a Gram-negative bacillus-shaped bacterium, which belongs to the Enterobacteriaceae family [80]. To date, 28 species have been identified, 3 of which are pathogenic to humans [81]. *Yersinia* spp. are heterogeneous species represented by six biotypes (1A, 1B, 2, 3, 4, 5) and different serogroups showing specific virulence factors correlated with the geographic region in which the bacteria occur [80]. This pathogen is mainly transmitted through raw food or water sources, causing gastrointestinal disease also known as yersinosis in humans. It is the fourth most frequently reported foodborne bacterial disease and a huge threat to human life [82]. The main symptoms of yersiniosis are fever, often hemorrhagic diarrhea, lymphadenitis, abdominal pain, and nausea [83]. Combating yersinosis is a great challenge since numerous representatives of this species have developed resistance to many frontline antibiotics such as penicillin, ampicillin, cephalosporine and macrolides [84]. Clearance of *Yersinia* contamination in food and food processing equipment is also difficult due to the ability of this pathogen to form biofilm structures [85].

Compared to phages specific to other foodborne pathogens, the knowledge regarding *Yersinia* phages is rather scarce. Jun et al. (2018) isolated and characterized four virulent bacteriophages specific to *Yersinia enterocolitica*. Bacteriophage fHe-Yen3-01 was assigned to the family Podoviridae, while three other phages (fHe-Yen9-01, fHe-Yen9-02 and fHe-Yen9-03) were assigned to the Myoviridae family. In subsequent tests, isolated viruses were used to reduce contamination in selected food products. Raw pork and milk were contaminated with the *Y. enterocolitica* O:9 Ruokola/71 strain, a genetically modified strain of *Yersinia* for use in in vitro tests. The bacterial count during the experiment decreased in raw pork from 2.3 × 10^3^ CFU/g to 2.12 × 10^2^ CFU/g and in milk from 4.15 × 10^3^ CFU/mL below the detection limit (<10 CFU/ml). In the case of RTE pork, the reduction of bacterial count was from 2.1 × 10^3^ CFU/g to 3.8 × 10 CFU/g. Furthermore, potential application of phages against *Yersinia* contamination on kitchen utensils, such as cutting boards, wooden spoons and kitchen knives, has been evaluated. Tools were immersed in a bacterial inoculum with the concentration of 10^4^ CFU/mL, then they were exposed to phages directed against *Y. enterocolitica*. The highest efficiency was demonstrated for the fHe-Yen9-01 phage, which reduced the number of bacteria by 1/3 compared to the initial bacterial count. These findings suggest potential use of phages to control the growth of *Y. enterocolitica* in food and everyday kitchen items [86].

*Yersinia* bacteriophages have also been used for the development of a selective tool for identification of this bacteria. Immune separation (IMS) was considered a promising approach in identifying *Yersinia* spp.; however, due to the existence of numerous serotypes of the pathogen, the method did not eventually find practical application. Therefore, many attempts have been made to modify this technique. One of them is based on use of magnetic microparticles together with RNA binding proteins (RBPs) Gp17, Gp47 and Gp37 of phages to selectively “capture” the epidemiological serotypes of *Y. enterolytica*. In the case of microparticles coated with RBP Gp17, it was possible to detect the O:3 type, which is the most virulent serotype causing yersiniosis. Moreover, use of Gp47 and Gp37 allowed for the identification of serotypes O:3, O:5, 27, O:8 and O:9. Notably, compared to the method based on antibodies, the modified IMS technique is stable to physical–chemical interactions [83]. The results confirm the potential of phages in the development of identification systems for different bacterial species, including food-related pathogens.

## 2. Perspectives

Microbial safety is one of the priority issues in the food industry as it directly affects consumers’ health. Currently applied antibacterial strategies have their limitations and thus solutions that are easy to implement, do not influence product quality, and are safe and cheap are eagerly anticipated. 

Bacteriophage effectivity against foodborne pathogens has been confirmed in numerous studies, and the potential of bacterial viruses in strategies against this group of bacteria has been noted by many scientists (Table 1) [42,60,87]. It is worth emphasizing that this review presents recent studies on the effectiveness of bacterial viruses in combating only selected food-related bacteria, and research studies in this field are much more advanced. They also include such other important foodborne pathogens as e.g., *Shigella* sp. [49,88], *Staphylococcus aureus* [89,90], *Pseudomonas* [91,92] and *Vibrio* [87].

Despite the fact that food safety is the industrial area in which commercialization of bacteriophage-based products shows the fastest progress, popularization of phage application is still in its infancy (Table 2). Over the last 12 years, the number of acceptable bacteriophage preparations approved for use in the food industry has increased. In 2006, the FDA issued the first approval for a bacteriophage cocktail against *L. monocytogenes* on RTA meat and poultry products, which was the aforementioned ListShield (LMP-102) (Intralytix, Columbia, USA). Later, approvals by the FDA were issued for other preparations such as PhageGuard and Listex (Micreos Food Safety B.V., Wageningen, The Netherlands), EcoShield (Intralytix, Columbia, USA), ShigaShield (Intralytix, Columbia, USA) and SalmoFresh (Intralytix, Columbia, USA). GRAS status was given to SalmoFresh (Intralytix, Columbia, USA) and PhageGuard (Micreos Food Safety B.V., Wageningen, The Netherlands) [93]. Currently 13 phage preparations for food safety applications have been approved by different North American or European institutions, most of them dedicated to fighting *E. coli* (6) and *Salmonella* spp. (4). Other preparations are active against *L. monocytogenes* (2) and *Shigella* spp. (1) [25]. It is worth noting a phage-based preparation dedicated for applications in agriculture, AgriPhage, offered by Omnilytix (USA), is active against *Xanthomonas campestris* pv. *vesicatoria* and *Pseudomonas syringae* pv. *tomato* and is available for sale [94]. Importantly, there are also preparations which have not yet received acceptance by western institutions but have been commercialized on the Asian market. As an example, BAFASAL (Proteon, Łódź, Poland), available as a feed additive and targeting *S.* Typhimurium and *S.* Enteritidis, still awaits commercialization on the European market [95].

Despite the many advantages of bacteriophage-based antibacterial strategies, there are also some drawbacks, which have to be considered particularly in the context of widespread phage application. One of the best known is the phage ability to randomly transfer fragments of the bacterial genetic material in the process called transduction [96]. Obviously transduction may include potentially harmful genes of virulence or antibiotic resistance contributing to the environmental spread of the strains of increased risk for public health. Clearly, this potential threat has to be considered with respect to intended application of bacterial viruses. 

Furthermore, the direct influence of phages on the human organism has not been comprehensively investigated. Notably, the assumption regarding safety of the use of phages results primarily from many years of clinical experience rather than scientific knowledge. Numerous research studies suggest the ability of bacterial viruses to interact with mammalian cells [97,98,99]; consequently, such mutual interactions should be thoroughly investigated.

As mentioned above, phage specificity is also a substantial limitation in widespread application of bacteriophages, particularly when food products are contaminated with different foodborne pathogens. However, this may be easily overcome by using a phage cocktail with a broad lytic spectrum and targeting different bacteria. Phage resistance may influence activity of a phage preparation, but including several phages with similar specificity into one cocktail should ensure activity of the product. From a purely practical point of view, isolation, propagation and application of phages for food safety are rather easy. Nevertheless, for development on a conventional product all the procedures have to be well established, standardized and repeatable. This, together with the extremely rigorous requirements concerning newly registered products, makes the pace of approval of commercial phage preparations still insufficient.

The question of whether commercialization of phage application in food safety can be expedited remains open. On one hand, there is an emerging global problem of drug resistance of many bacterial strains which demands new solutions for fighting microbial contamination. This issue also naturally concerns the food industry. On the other hand, we are at a point in history when mankind is about to face a major food crisis. Improving the safety of food and thus safeguarding it from wastage seems to be a critical challenge. Undoubtedly, multifaceted application of bacteriophages in combating foodborne pathogens is an important option.

## 3. Conclusions

Food products are one of the main routes of transmission of infectious diseases throughout the human population. Therefore, effective, safe and easily implemented methods ensuring protection of consumers are highly desired.

Bacteriophages have properties that make them excellent candidates in antibacterial approaches, especially in the food safety sector. Numerous studies confirm the high efficiency of phages in eliminating various foodborne pathogens. This includes drug-resistant bacteria and highly stable biofilm structures, both representing a particular concern for food producers. At the same time, application of phages does not influence the quality of food products and is easy and safe. Although commercialization of phage-based products in food safety has been steadily progressing, much still needs to be done to achieve widespread use of phage preparations. Nevertheless, considering the limitations and nearly exhausted potential of currently applied antibacterial methods, new strategies are highly desirable. Undoubtedly, application of phage products may be considered an attractive choice.

## Figures and Tables

**Table 1:** Exemplary studies on bacteriophage use against selected foodborne pathogens.

Bacteria	Symptoms	Transmission	Phages	Results	References
*Campylobacter jejuni*	Fever, muscle aches, headaches, arthralgia, abdominal pain and cramps, weakness, bloody diarrhea, gastric or intestinal pain, occurrence of Guillain-Barré syndrome	Poultry meat, milk, contaminated water, swimming in contaminated water bodies, contact with animal.	CJ01	Mutton and chicken meat were stored at 4 °C and injected with 5 mL of *C. jejuni* with a concentration of 10^4^ CFU/mL. The samples prepared in this way were incubated for 4 h. Then, they were sprayed with 5 mL of bacteriophage with PFU/mL, and the samples were again incubated for 48 h. The final result was 10^2^ CFU/g.	[4]
			Φ7-izsam Φ16-izsam	The influence of bacteriophages on naturally or artificially contaminated poultry was investigated. Bacteriophages were given to the animals before slaughter and resulted in a reduction of 1 log10 CFU/g and 2 log10 CFU/g for both test groups.	[72]
			CP20 and CP30A	Poultry were infected and bacteriophages were administered 4 days later. Chickens were sacrificed every 24 h and the intestinal pathogen concentration was examined. The most prominent result was obtained on the second day of incubation and caused a decrease of bacteria by 2.4 log CFU/g in relation to the control.	[73]
			12673, P22, 29C;	The contaminated skin of chickens was examined. The level of the pathogen decreased by 2 log units when using the MOI of the phage 100:1 or 1000:1.	[100]
*Escherichia coli*	Vomiting, headaches, stomach pain, low-grade fever or fever, diarrhea, weakness, bloody stools, hemolytic uremic syndrome, neonatal meningitis, pneumonia, sepsis	Pork, poultry, contaminated ruminants such as goats, deer, sheep, elk, water, milk and dairy products, direct contact with animals	FM10, DP16 and DP19	The phage cocktail was tested on fresh intubated cucumber at two temperatures of 4 °C and 25 °C for 24 h. The number of bacteria was reduced by 1.97–2.01 log CFU/g and 1.16–2.01 log CFU/g at 25 °C and 4 °C.	[54]
			DW-EC	It was tested on many matrices, such as chicken meat, lettuce meat, fish meat, and tomato. The samples were contaminated with bacteria, then they were subjected to the phage section. A significant result was obtained at 6 days of incubation. The best effect was seen in the chicken feed samples where the pathogen value decreased by 80.93% after the first day and 87.29% after the 6th day. The weakest effect was observed on lettuce leaves.	[55]
			AZO145A	The effect of phage on the biofilm was investigated. Exposure to bacteriophage at a concentration of 10^10^ PFU/cm^2^ for 2 h resulted in a 4.0 log 10 PFU/mL reduction in biofilm on stainless steel. However, on the surface of beef, at 48 h incubation, the pathogen decreased by 3.1 log10 CFU/g.	[56]
			T4	The aim of the study was to design an antimicrobial package by using the immobilization of T4 phage (10^5^ CFU/mL) on the surface of the PCL foil. Contaminated beef was placed in this package. The bacterial concentration applied to the meat was 10^7^ CFU/mL. After 48 h of incubation, the concentration of bacteria was reduced by 3 log CFU/mL.	[84]
			FAHEc1	Contaminated raw beef as a test matrix; after using phage, the concentration of bacteria decreased by 2 or 4 log units at the appropriate storage temperatures, 24 °C and 37 °C.	[101]
*Listeria monocytogenes*	Fever, chills, muscle aches, headaches, nausea and vomiting, confusion, local infections, inflammation of the lymph nodes, inflammation of the lungs, joints, bone marrow, pericarditis and myocarditis, inflammation of the eyeball, gastrointestinal infections.	Raw vegetables and fruit, unpasteurized dairy products (milk, cheese, ices cream), raw, cooked and frozen poultry meat, raw and smoked fish, delicatessen products, semi-finished products, fast-food products, soil, sewage, water, rotting plants, silage, wild and farm animals.	FWLLm1	Bacterial levels dropped by 2 log units on the surface of the chicken that had become contaminated with *Listeria*. The samples were stored in a vacuum package at 4 °C and 30 °C. A positive result was observed only for the sample kept at 30 °C.	[7]
			A511	Bacterial levels were tested in milk chocolate, mozzarella and brie cheese. The phage were given and incubated at 6 °C. Bacteria concentration dropped by 5 log units.	[102]
*Pseudomonas* spp.	Pneumonia, fever, chills, severe shortness of breath, cough, confusion, chronic lower respiratory tract infection, Roth’s spots, i.e., petechiae on the retina, small painless erythematous changes on the hands and feet—Janeway symptom, painful reddish lumps on the fingers—Osler’s nodules, subungual petechiae	Water, soil, human and animal digestive tract.	UFJF_PfDIW6, UFJF_PfSW6	The lyophilized phage cocktail was incubated with raw milk at 4 °C for 7 days. After the incubation period, the *Pseudomonas* bacterial population decreased by 3.2 log CFU/mL.	[103]
			V523, V524, JG003	Three bacteriophages used separately and together as a cocktail were used to biocontrol bacteria in the water. The effect of the phages was tested against two bacteriophage strains: PAO1 and the environmental strain 17V1507. Of all the bacteriophages, V523 was most effective in reducing the PAO1 strain (>2.4 log10). The other strain was sensitive only to JG003, resulting in its reduction by 1.2 log10. The phage cocktail resulted in higher reductions in PAO1 (>3.4 log10) compared to using them alone. In contrast, the same reduction was observed in 17V1507 as with JG003 alone.	[104]
*Salmonella* spp.	Abdominal pain, vomiting, diarrhea, fever, headache, chills, reduced urine output, dry mucous membranes, excessive sleepiness, apathy.	Chicken, turkey, pig, duck, goose meat, eggs, soil, water, cheese, milk, fruit, vegetables, contact with contaminated animals	LPSTLL, LPST94, LPST153	The use of the bacteriophage cocktail decreased the concentration of bacteria by 3 log units. Chicken breasts were inoculated using an inoculum. The influence on the biofilm created by *Salmonella* was also examined, the administered cocktail effectively inhibited the growth after 72 h, the microplates decreased by 5.23 log units.	[46]
			LYPSET	The biocontrol was tested in milk and on lettuce leaves. In milk samples there was a decrease of bacteria by 2.19 log CFU/mL at 4 °C and 4.3 log CFU/mL at 25 °C. However, in the samples containing lettuce, there was a decrease of 2.2 log CFU/mL at 4 °C and 2.34 log CFU/mL at 25 °C at MOI = 10,000.	[45]
			SE07	The effects of phage on eggs, beef and poultry meat were tested. The best effect for beef was obtained after 48 h of incubation; the bacterial value dropped from 4.23 log CFU/mL to 2.11 log CFU/mL, while for chicken the effect was even better as there was a decrease from 4.16 log CFU/mL to 2.14 log CFU/mL also at 48 h.	[47]
			SJ2	The use of phage resulted in a significant reduction of bacteria in the soft pork and eggs. Incubation was carried out at 4 °C.	[105]
			BSPM4, BSP101, BSP22A	The phages were presented as a cocktail. The reduction of bacterial colonies was tested on lettuce leaves and fresh cucumber. There was a reduction of 4.7 log for lettuce and 5.8 log for cucumber.	[106]
*Shigella* sp.	Vomiting, anorexia, abdominal cramps, bowel urgency, severe watery diarrhea, fever, diarrhea with an admixture of mucus and blood, rapid breathing, heart rate, low blood pressure, dry mouth and skin (dehydration), pain on palpation of the abdomen	Touching skin of contaminated person, oral cavity (fecal–oral route), contaminated water and food, sexual contact, swimming in contaminated water, by insects, such as housefly.	SSE1, SGF3, SGF2,	The influence of the SFE3 phage and its combination as a cocktail with other phages on the reduction of biofilm on polystyrene surfaces was investigated. It was found that the single SGF2 phage (isolated from wastewater) had the greatest impact on the development of biofilm; it caused growth inhibition by 26.6%. The lowest results were obtained for SGF3, while adding it to a phage cocktail increased its effectiveness by 25%. The phage is active against strains such as *S. dysenteriae*, *S. baumannii*, and *S. flexneri*.	[107]
			SD-11, SF-A2, SS-92	The number of pathogens was decreased by 4 log in chicken meat, when they applied phage cocktail. It was stored at 4 °C.	[108]
*Staphylococcus* sp.	Infections of the skin and subcutaneous tissue, which are characterized by the presence of purulent discharge, impetigo, folliculitis, boils, furunculosis (multiple boils), abscesses, inflammation of the sweat glands and inflammation of the mammary gland, high fever, drop in blood pressure, organ dysfunction	Transmission mainly by direct contact. Patients after surgery are most at risk	MDR, ME18, ME126	Reducing biofilm in UHT milk at 25 °C using ME18 (MOI = 10) and MDR. They reduce biofilm in milk. However ME126 (MOI = 10) at 37 °C reduces CFU/mL by 87.2% compared to control sample.	[109]
*Vibrio parahaemolyticus*	Watery diarrhea, abdominal cramps, nausea, vomiting, fever or chills, abscess formation, otitis media, otitis media and conjunctivitis	Contact with contaminated water, fruit, seafood	PVP1 and PVP2	They treated sea cucumber contaminated with pathogen. MOI = 10 or MOI = 100. Test were performed at in 20 °C and it increased survival of sea cucumber to 80% compared with control sample without phage cocktail treatment, which was only 30%.	[110]
*Yersinia*	Mild or high fever, cramping abdominal pain, loose stools often with mucus or blood, vomiting, right-hand stomach pain, tenderness when examining the abdominal cavity, fast heartbeat, joint pains, mainly in the knee, ankle and wrist, rapid breathing	Pork and pork offal, milk, water, raw vegetables and fruits.	fHe-Yen3-01 fHe-Yen9-01, fHe-Yen9-02 and fHe-Yen9-03	Infected raw pork and cooking tools with the Rukola/71 strain. The kitchen tools were immersed in an inoculum at a concentration of 10^4^ CFU/mL. The best effect was obtained for the phage fHe-Yen9-01, which reduced the number of bacteria by 1/3.	[86]
			PY100	The given phage reduced the amount of bacteria in the meat MOI = 10^2^ by 3 log10 units after 24 h incubation and at a MOI = 10^4^ by 5 log10 units after 1.5 h incubation at 37 °C. However, when incubated at 4 °C, the bacteria count decreased by 2 log units after 24 h.	[111]

**Table 2:** Selected commercial bacteriophage preparations.

Company	Product	Target	Reference	Regulatory & Certifications
Micreos Food Safety (The Netherlands)	PhageGuard Listex	*Listeria* sp.	[65,112,113]	Halal, OMRI, Kosher, Skal, FSSC 2200; FDA, GRN 198/21, EFSA; Swiss BAG; Israel Ministry of Health; Health Canada
	PhageGuard S	*Salmonella enterica*	[114]	Halal, FSSC 22000; FDA, GRN 468; USDA, FSIS Directive 7120.1, Swiss BAG
	PhageGuard E	*Escherichia coli* O157:H7	-	FSSC 22000
Intralytix (USA)	ListShield	*Listeria monocytogenes*	[3,66,115,116,117]	Kosher; Halal; OMRI; FDA, 21 CFR 172.785; FDA, GRN 528;
	SalmoFresh	*Salmonella enterica*	[5,49,118,119,120]	Kosher; Halal; OMRI FDA, GRN 435; USDA, FSIS Directive 7120.1
	ShigaShield	*Shigella* sp.	[88,121,122]	FDA, GRN 672
	EcoShield PX	*Escherichia coli*	[9,123,124,125]	FDA, GRN 834; USDA, FSIS Directive 7120.1
	CampyloShield	*Campylobacter* spp.	[126]	GRAS
Proteon Pharmaceuticals SA (Poland)	Bafasal	*Salmonella enterica*	[96,127]	-
	Bafador	*Pseudomonas* sp., *Aeromonas* sp.	-	-
Passport Food Safety Solutions	Finalyse	*E. coli O157:H7*	-	USDA, FSIS Directive 7120.1
Phagelux	SalmoPro	*Salmonella* spp.	-	FDA, GRN 603; USDA
FINK TEC GmbH (Hamm, Germany)	Secure Shield E1	*E. coli*	[93]	FDA,GRN 724
Arm and Hammer Animal & Food Production (USA)	Finalyse SAL	*Salmonella*	[128]	-

## Data Availability

Not applicable.
